# Supplementary material for: SARS-CoV-2 Simulations Go Exascale to Capture Spike Opening and Reveal Cryptic Pockets Across the Proteome
Source: Nat Chem. Author manuscript; Available in PMC 2021 Jul 2. (PMC8249329; doi:10.1038/s41557-021-00707-0)
Supplement: 1 [file NIHMS1714208-supplement-1.pdf]

bioRxiv preprint doi: https://doi.org/10.1101/2020.06.27.175430; this version posted October 7, 2020. The copyright holder for this preprint (which was not certified by peer review) is the author/funder, who has granted bioRxiv a license to display the preprint in perpetuity. It is made available under aCC-BY 4.0 International license.

Supporting Information to SARS-CoV-2 Simulations Go Exascale to Capture Spike Opening and Reveal Cryptic Pockets Across the Proteome

Maxwell I. Zimmerman[1,2], Justin R. Porter[1,2], Michael D. Ward[1,2], Sukrit Singh[1,2], Neha Vithani[1,2], Artur Meller[1,2], Upasana L. Mallimadugula[1,2], Catherine E. Kuhn[1,2], Jonathan H. Borowsky[1,2], Rafal P. Wiewiora[3,4], Matthew F. D. Hurley[5], Aoife M Harbison[6], Carl A Fogarty[6], Joseph E. Coffland[7], Elisa Fadda[6], Vincent A. Voelz[5], John D. Chodera[4], Gregory R. Bowman[1,2,]*

[1] *Department of Biochemistry and Molecular Biophysics, Washington University School of Medicine, St. Louis, Missouri 63110, United States*

[2] *Center for Science and Engineering of Living Systems (CSELS), Washington University in St. Louis, St. Louis, Missouri 63130, United States*

[3] *Tri-Institutional PhD Program in Chemical Biology, Memorial Sloan Kettering Cancer Center, New York, New York 10065, United States*

[4] *Computational and Systems Biology Program, Sloan Kettering Institute, New York, New York 10065, United States*

[5] *Department of Chemistry, Temple University, Philadelphia, Pennsylvania 19122, United States*

[6] *Department of Chemistry and Hamilton Institute, Maynooth University, Maynooth, Kildare, Ireland*

[7] *Cauldron Development LLC*

**Corresponding Author: g.bowman@wustl.edu*

bioRxiv preprint doi: https://doi.org/10.1101/2020.06.27.175430; this version posted October 7, 2020. The copyright holder for this preprint (which was not certified by peer review) is the author/funder, who has granted bioRxiv a license to display the preprint in perpetuity. It is made available under aCC-BY 4.0 International license.

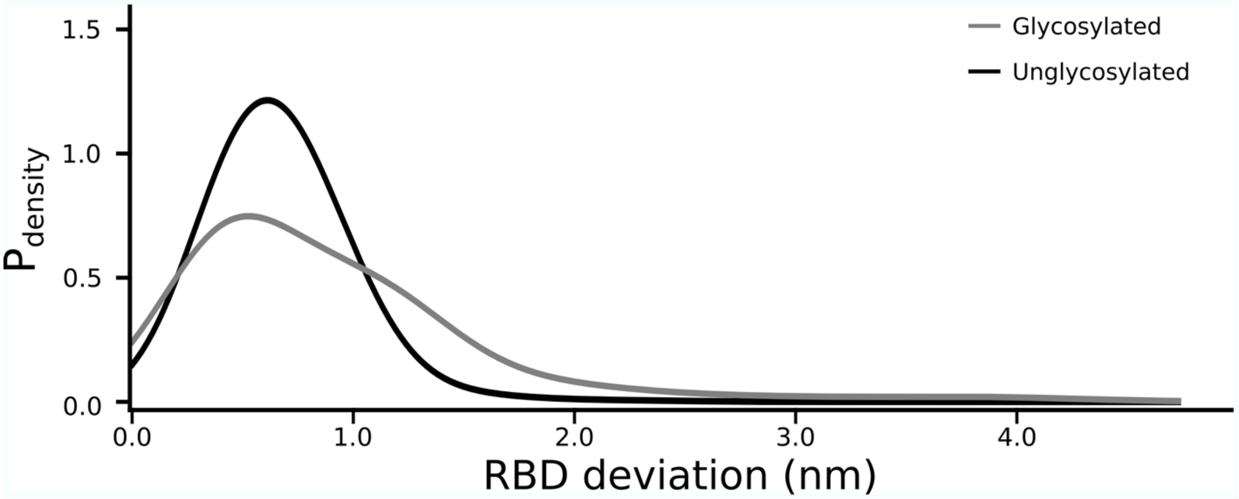

Figure S1: Distribution of SARS-CoV-2 Spike RBD opening. The probability that the center of mass of an RBD deviates from its position in the closed (or down) state for SARS-CoV-2 spike with glycans (gray) and without glycans (black).

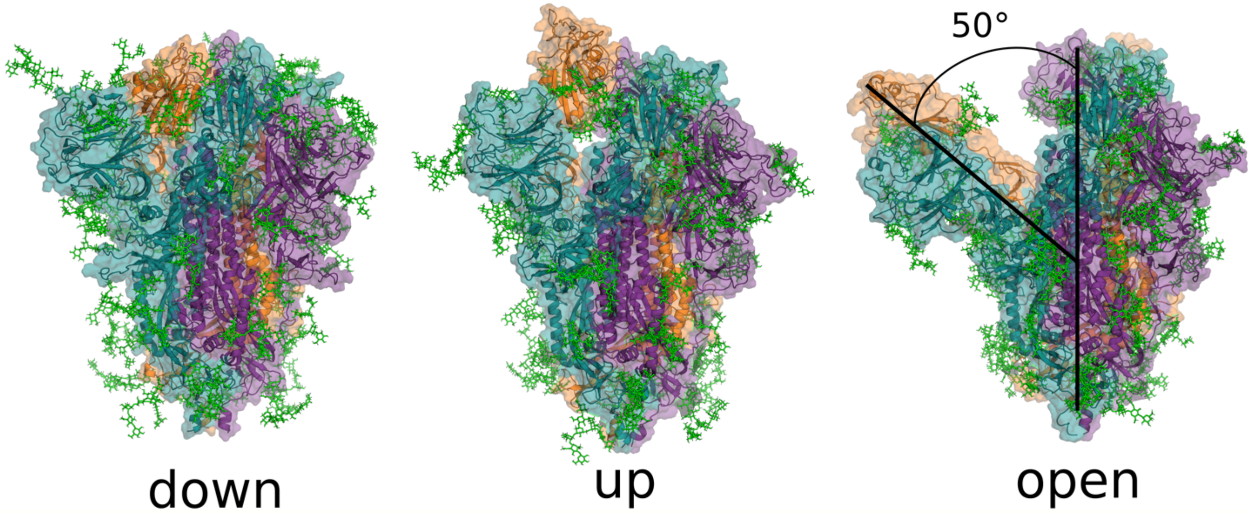

Figure S2: Simulations of the SARS-CoV-2 Spike complex reveal the existence of an “open” state. For reference, three Spike complex snapshots are shown: the “down” state (6VXX), the “up” state (6VSB), and an “open” state from our simulations. Structures are depicted with a cartoon backbone, transparent surface for sidechains, and sticks for glycans. Each chain in the complex has a unique color, orange, purple, or teal, and glycans are colored green.

bioRxiv preprint doi: https://doi.org/10.1101/2020.06.27.175430; this version posted October 7, 2020. The copyright holder for this preprint (which was not certified by peer review) is the author/funder, who has granted bioRxiv a license to display the preprint in perpetuity. It is made available under aCC-BY 4.0 International license.

Cryptic pocket highlights for select systems in the SARS-CoV-2 proteome

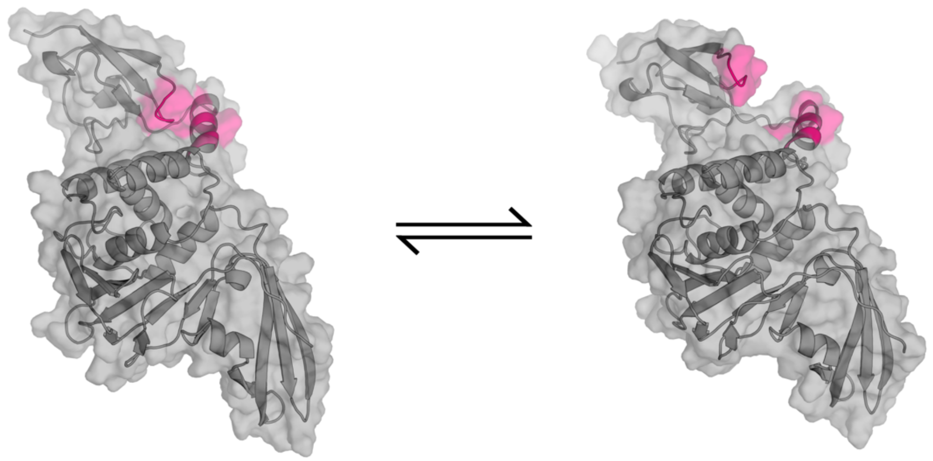

Figure S3: NSP3-PL2Pro domain transition from closed to open state. Backbone is represented as a cartoon and sidechains are represented with a transparent surface (gray). The residues that undergo a large conformational change to expose a cryptic pocket are highlighted in pink.

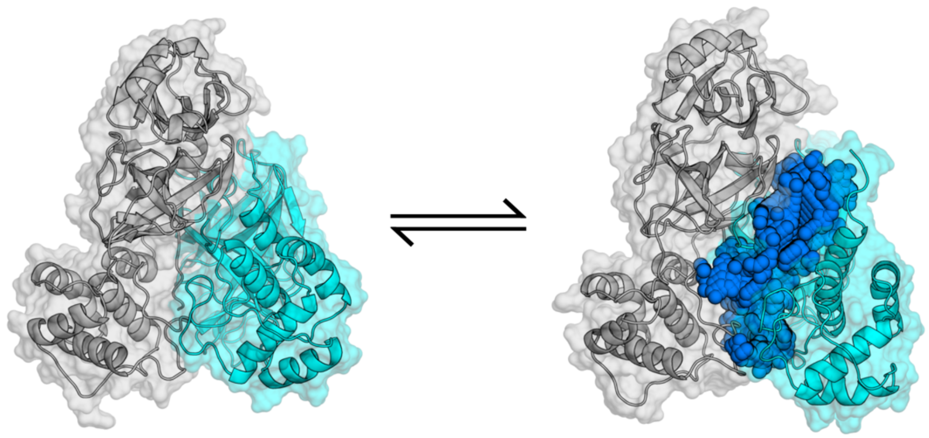

Figure S4: NSP5 (dimer) transition from closed to open state. Backbone is represented as a cartoon, sidechains are represented with a transparent surface, and pocket volumes are represented as blue spheres. Each molecule in the dimer is identified with a unique color, gray or cyan.

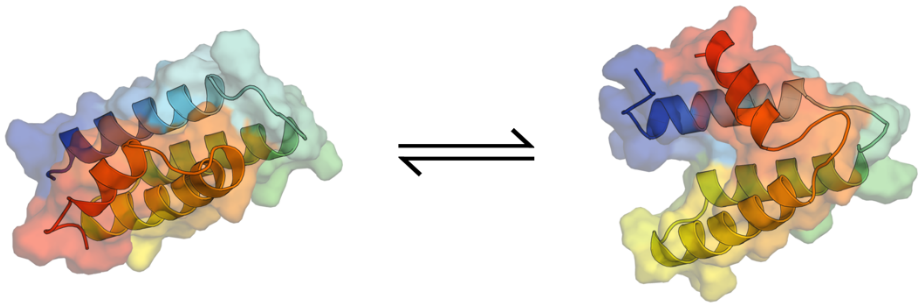

Figure S5: NSP7 transition from closed to open state. Backbone is represented as a cartoon and sidechains are represented with a transparent surface. The protein is colored by residue number following a rainbow.

bioRxiv preprint doi: https://doi.org/10.1101/2020.06.27.175430; this version posted October 7, 2020. The copyright holder for this preprint (which was not certified by peer review) is the author/funder, who has granted bioRxiv a license to display the preprint in perpetuity. It is made available under aCC-BY 4.0 International license.

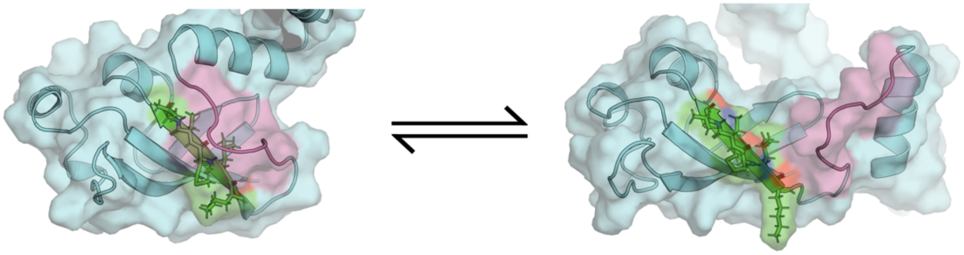

Figure S6: NSP8 transition from closed to open state. Backbone is represented as a cartoon and sidechains are represented with a transparent surface. For reference, two regions that undergo a large conformational transition are highlighted as green and pink.

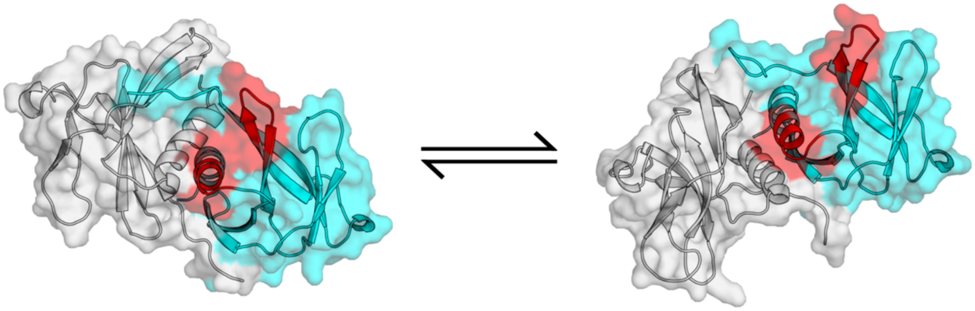

Figure S7: NSP9 (dimer) transition from closed to open state. Backbone is represented as a cartoon and sidechains are represented with a transparent surface. Each molecule in the dimer is identified with a unique color, gray or cyan. The residues that undergo a large conformational change to expose a cryptic pocket are highlighted in red.

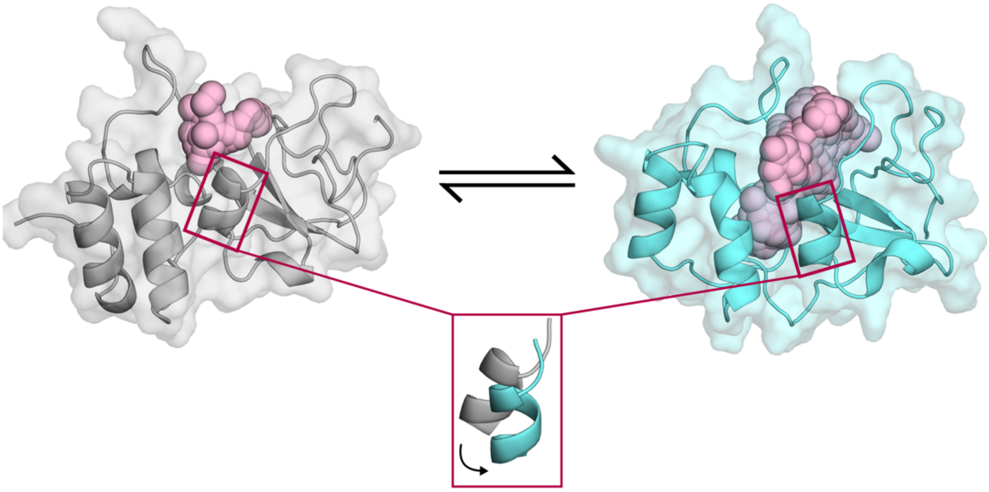

Figure S8: NSP10 transition from closed to open state. Backbone is represented as a cartoon and sidechains are represented with a transparent surface. Pocket volumes are highlighted with pink spheres. Here, an existing pocket is greatly expanded from the swivel of an α-helix.

bioRxiv preprint doi: https://doi.org/10.1101/2020.06.27.175430; this version posted October 7, 2020. The copyright holder for this preprint (which was not certified by peer review) is the author/funder, who has granted bioRxiv a license to display the preprint in perpetuity. It is made available under aCC-BY 4.0 International license.

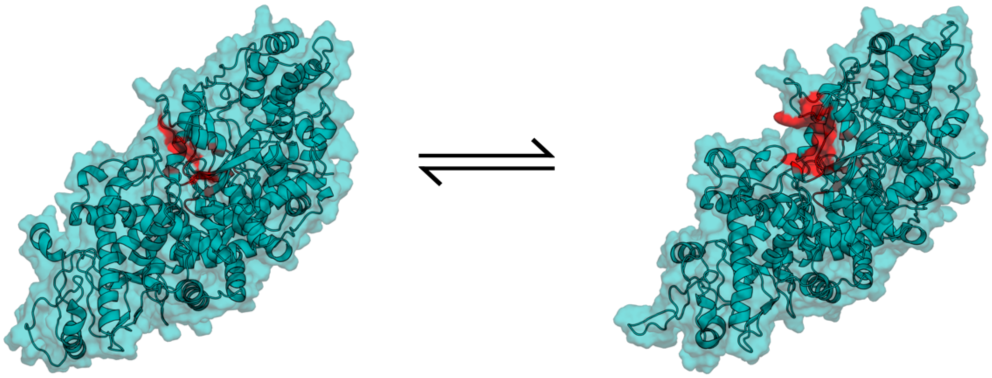

Figure S9: NSP12 transition from closed to open state. Backbone is represented as a cartoon and sidechains are represented with a transparent surface. The residues that undergo a large conformational change to expose a cryptic pocket are highlighted in red.

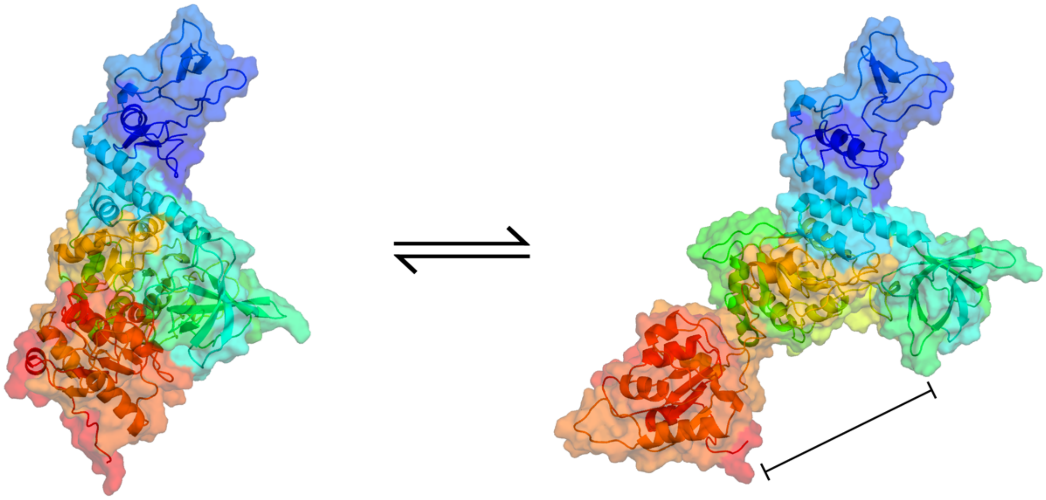

Figure S10: NSP13 transition from closed to open state. Backbone is represented as a cartoon and sidechains are represented with a transparent surface. The protein is colored by residue number following a rainbow and highlights the various domains. Here, we observe a large domain motion between domains 1A and 2A, which may be relevant for nucleotide binding.

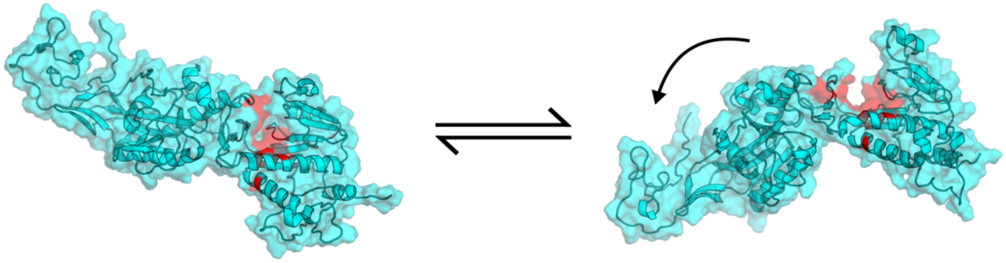

Figure S11: NSP14 transition from closed to open state. Backbone is represented as a cartoon and sidechains are represented with a transparent surface. The residues that undergo a large conformational change to expose a cryptic pocket are highlighted in red.

bioRxiv preprint doi: https://doi.org/10.1101/2020.06.27.175430; this version posted October 7, 2020. The copyright holder for this preprint (which was not certified by peer review) is the author/funder, who has granted bioRxiv a license to display the preprint in perpetuity. It is made available under aCC-BY 4.0 International license.

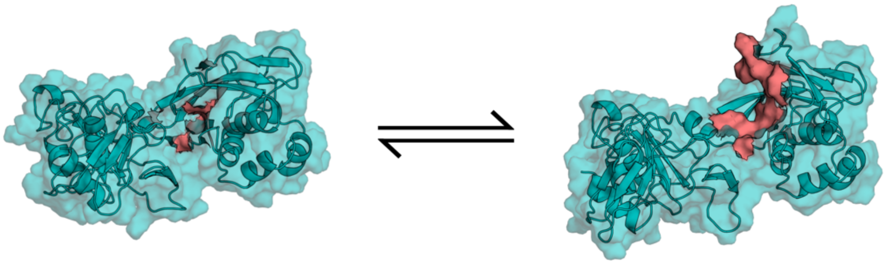

Figure S12: NSP15 transition from closed to open state. Backbone is represented as a cartoon and sidechains are represented with a transparent surface. The residues that undergo a large conformational change to expose a cryptic pocket are highlighted in red.

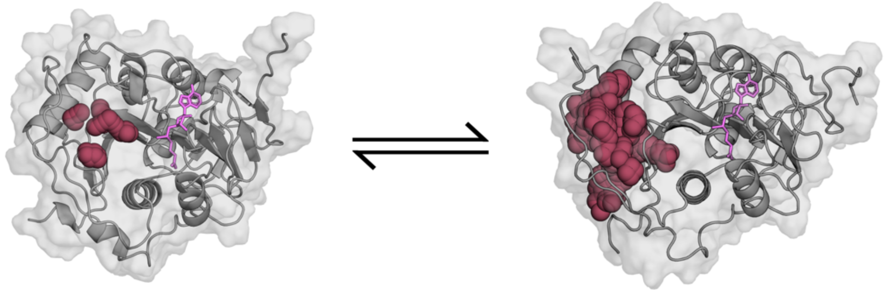

Figure S13: NSP16 transition from closed to open state. Backbone is represented as a cartoon and sidechains are represented with a transparent surface. Pocket volumes are highlighted with maroon spheres. SAM cofactor is shown with pink sticks.

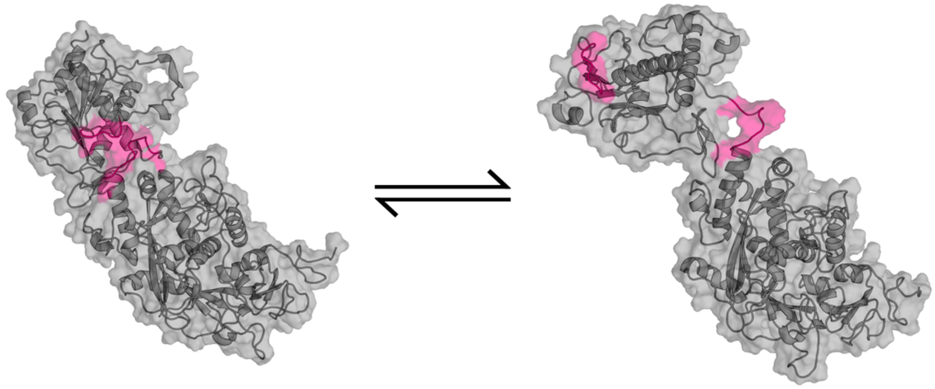

Figure 5: NSP10/NSP14 (complex) transition from closed to open state. Backbone is represented as a cartoon and sidechains are represented with a transparent surface. The residues that undergo a large conformational change to expose a cryptic pocket are highlighted in pink.

bioRxiv preprint doi: https://doi.org/10.1101/2020.06.27.175430; this version posted October 7, 2020. The copyright holder for this preprint (which was not certified by peer review) is the author/funder, who has granted bioRxiv a license to display the preprint in perpetuity. It is made available under aCC-BY 4.0 International license.

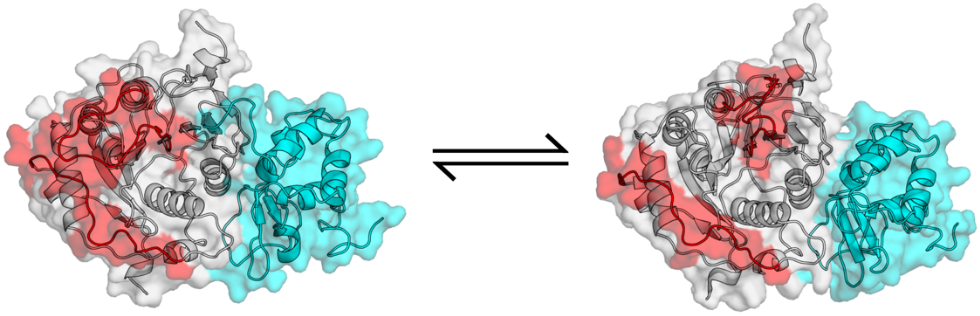

Figure S15: NSP10/NSP16 (complex) transition from closed to open state. Backbone is represented as a cartoon and sidechains are represented with a transparent surface. Each molecule in the complex is identified with a unique color, gray (NSP16) or cyan (NSP10). The residues that undergo a large conformational change to expose a cryptic pocket are highlighted in red.

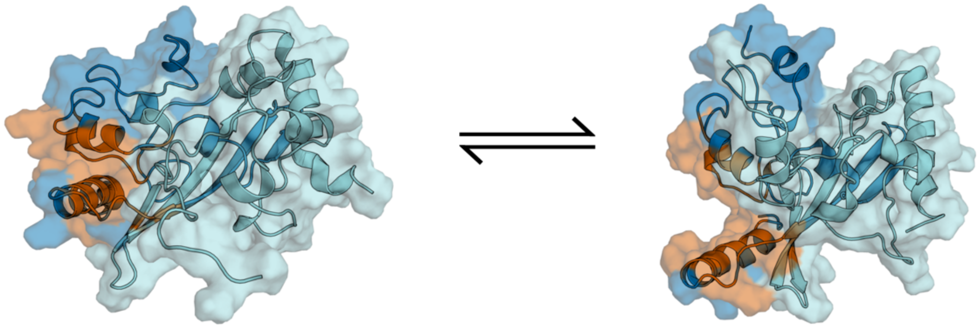

Figure S16: Nucleoprotein dimerization domain transition from closed to open state. Backbone is represented as a cartoon and sidechains are represented with a transparent surface. The residues that undergo a large conformational change to expose a cryptic pocket are highlighted in orange.

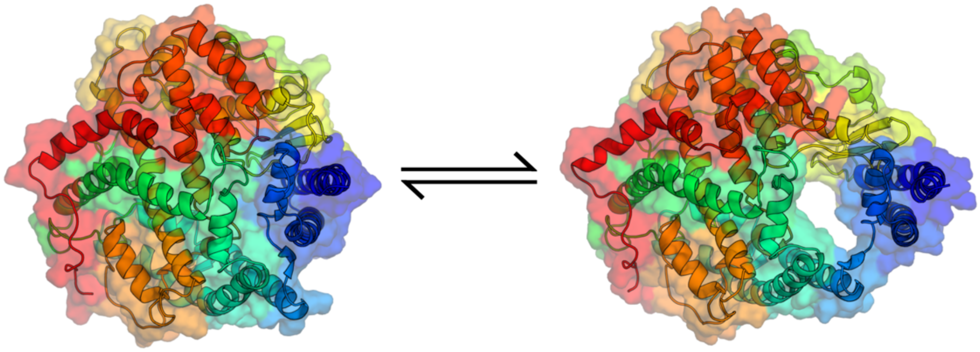

Figure S17: Human ACE2 transition from closed to open state. Backbone is represented as a cartoon and sidechains are represented with a transparent surface. The protein is colored by residue number following a rainbow. Pocket is proximal to the region that binds to SARS-CoV-2 spike protein.

bioRxiv preprint doi: https://doi.org/10.1101/2020.06.27.175430; this version posted October 7, 2020. The copyright holder for this preprint (which was not certified by peer review) is the author/funder, who has granted bioRxiv a license to display the preprint in perpetuity. It is made available under aCC-BY 4.0 International license.

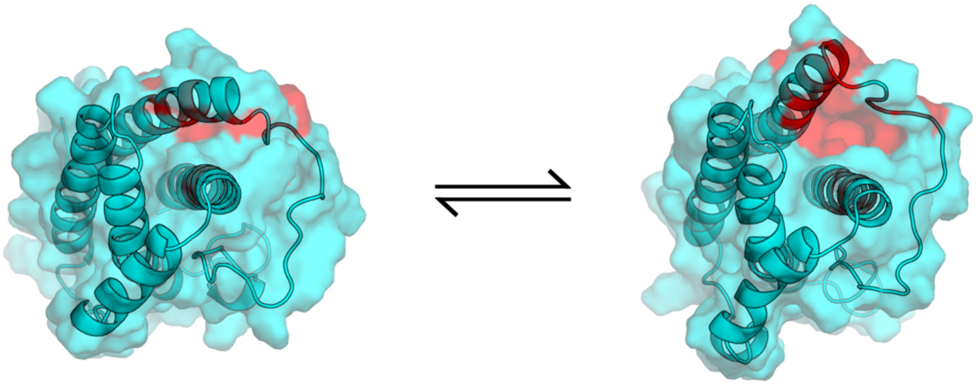

Figure S18: Human IL6 transition from closed to open state. Backbone is represented as a cartoon and sidechains are represented with a transparent surface. The residues that undergo a large conformational change to expose a cryptic pocket are highlighted in red.

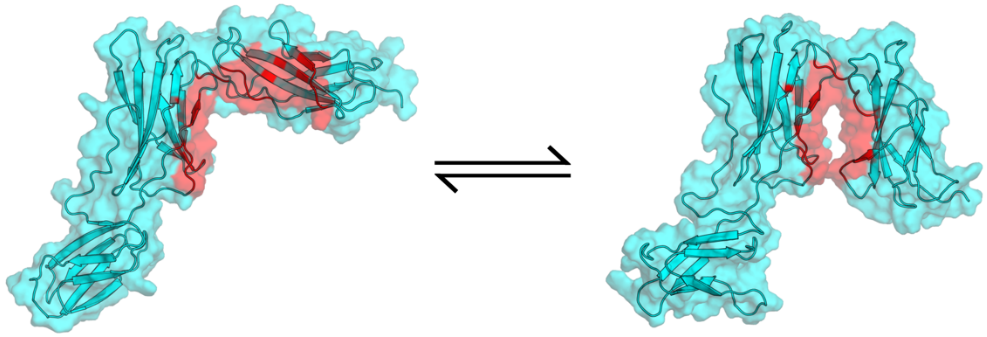

Figure 6: Human IL6-R transition from expanded to closed state. Backbone is represented as a cartoon and sidechains are represented with a transparent surface. The residues that undergo a large conformational change to reveal a potential druggable site are highlighted in red.
